# miR-185 inhibits cell migration and invasion of hepatocellular carcinoma through CDC42

**DOI:** 10.3892/ol.2019.11186

**Published:** 2019-12-04

**Authors:** Qingjun Zhang, Yun Chen, Ke Liu

Oncol Lett 16: 3101-3107, 2018; DOI: 10.3892/ol.2018.8971

Following the publication of the above article, the authors have drawn to our attention that Fig. 2 was erroneously printed in the paper twice: The first time correctly as Fig 2, but the same figure was subsequently printed as Fig. 3. The correct version of Fig. 3, as it should have appeared in this article, is shown opposite. This error was introduced during the pre-press stages, as the proofs of the article were being compiled. The Editor sincrely apologizes to the authors for this mistake, and we regret the inconvenience and confusion that this has caused.

## Figures and Tables

**Figure 3.**
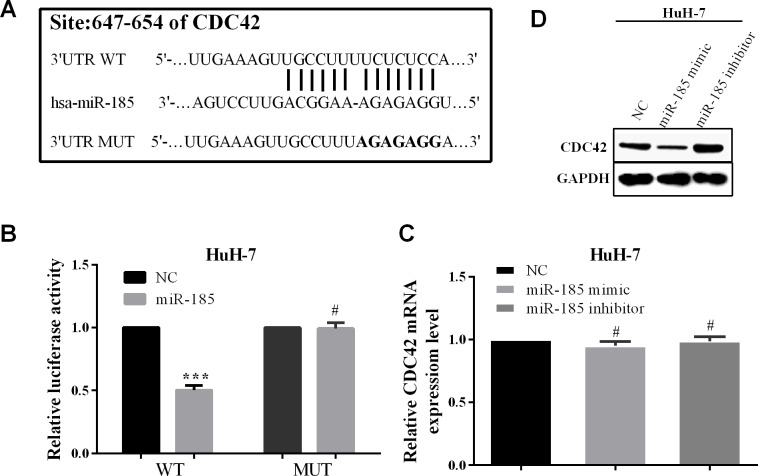
CDC42 is a target of miR-185 and mediated by miR-185. (A) The binding sites of CDC42 for miR-185 are located at the 3’-UTR. The mutated nucleotides are indicated by overstriking, and named ‘MUT’. (B) Changes of luciferase activity when cells were co-transfected with miR-365 or NC, and CDC42 3’-UTR WT or MUT. (C) The mRNA and protein level of CDC42 when transfected with miR-185 mimic or miR-185 inhibitor in HuH-7 cells. ***P<0.05; ^#^P>0.05. CDC42, cell division cycle 42. NC, negative control.

